# Spatial and Temporal Variation in the Antagonistic and Mutualistic Interactions among Seed Predator Arthropods, Seed-Dispersing Birds, and the Spanish Juniper

**DOI:** 10.3390/insects15080620

**Published:** 2024-08-18

**Authors:** Eduardo T. Mezquida, José Miguel Olano

**Affiliations:** 1 Department of Ecology, Faculty of Sciences, Autonomous University of Madrid, 28049 Madrid, Spain; 2 Biodiversity and Global Change Research Center (CIBC-UAM), Autonomous University of Madrid, 28049 Madrid, Spain; 3 Instituto Universitario de Investigación en Gestión Forestal Sostenible (iuFOR), Escuela de Ingeniería de la Industria Forestal, Agronómica y de la Bioenergía (EiFAB), Universidad de Valladolid, 42004 Soria, Spain; josemiguel.olano@uva.es

**Keywords:** cone traits, crop size, dispersal, frugivory, *Juniperus thurifera*, plant–animal interactions, predator satiation, predispersal seed predation

## Abstract

**Simple Summary:**

Plants interact with various animals during reproduction, which influences their success and evolution. Predispersal seed predators reduce fruit crop, potentially decreasing seed dispersal success through a reduction in the quantity and quality of fruits available for dispersers. We explored this phenomenon in a gymnosperm producing fleshy cones, Spanish juniper (*Juniperus thurifera*). Spanish juniper cones are damaged by three predispersal arthropod taxa and dispersed by birds. We assessed how these interactions change over time and across different locations. Cone production was the main factor affecting predispersal seed predation. High crops reduced relative seed predation rates due to a satiation effect, although seed dispersal did not increase significantly. Cone crop size had a strong effect on the relative abundance of each arthropod species, with higher competition among arthropods in years with low cone production. Crop size and cone traits at individual trees influenced which seed predators were attracted and the foraging activity of birds. Competition among arthropods increased during years with fewer cones, and seed predators sometimes deterred birds from eating the cones. Overall, large year to year fluctuations in cone production appear to favor Spanish juniper by reducing the impact of seed predators.

**Abstract:**

Plants interact with both antagonistic and mutualistic animals during reproduction, with the outcomes of these interactions significantly influencing plant reproductive success, population dynamics, and the evolution of plant traits. Here, we investigated the spatial and temporal variations in the interactions between *Juniperus thurifera*, its seed-dispersing birds, and three specific arthropod species that attack the fleshy cones during the predispersal period. We assessed how plant traits affect levels of cone damage by arthropods and seed dispersal by birds, the occurrence of competition among arthropod species, and the impact of seed predators on the activity of frugivores. Plant traits, cone damage by arthropods, and seed dispersal by birds showed spatiotemporal variability. Fluctuation in cone abundance was the leading factor determining damage by arthropods and bird dispersal with a secondary role of cone traits. Large crops satiated predispersal seed predators, although the amount of frugivory did not increase significantly, suggesting a potential satiation of bird dispersers. Crop size and cone traits at individual trees determined preferences by seed predator species and the foraging activity of bird dispersers. Competition among arthropods increased during years of low cone production, and seed predators sometimes negatively affected bird frugivory. High supra-annual variations in cone production appear to be a key evolutionary mechanism enhancing *J. thurifera* reproductive success. This strategy reduces the impact of specialized seed predators during years of high seed production, despite the potential drawback of satiating seed dispersers.

## 1. Introduction

Plants interact with multiple species at different times of their life cycle [1]. Antagonistic animals feed on different plant reproductive parts, exerting a negative impact on plant reproductive output [2,3,4]. On the other hand, mutualistic pollinators or seed dispersing animals play key roles in plant sexual reproduction and the dispersal of ripened seeds away from the parent plant, thus improving plant reproductive success [5,6,7,8]. The net outcome of these opposing interactions on plant reproductive performance will depend on multiple factors such as plant reproductive investment, plant traits, the strength of antagonistic and mutualistic interactions, and the potential direct or indirect effects among interacting animal species [9,10,11,12,13]. Ultimately, plant–animal interactions could determine seed output, seedling recruitment, and plant population dynamics [3,14,15,16] and can have important consequences for selection on plant reproductive traits [10,17,18,19].

Plant–animal interactions are sometimes relatively consistent in space, at least for some populations, or temporally when interactions involve few species or the core species that interact most frequently with each other in more complex species interactions [10,17,18,19,20,21,22]. However, biotic interactions are usually dynamic across space and time because of variation in abiotic conditions [23,24], the occurrence or abundance of interacting species [5,25,26], or the community context, including competitors, predators, or parasites that affect interacting species [27,28,29]. Spatiotemporal variations in species interactions might thus have important consequences for the spatial structure, population dynamics, and life history traits of the interacting species [30,31,32].

Plants that use mutualistic vertebrates as vectors to disperse their seeds produce fleshy structures covering the seeds as food rewards for frugivorous [33]. Several plant and fruit traits are generally associated with higher fruit consumption by vertebrate frugivores and thus seed dispersal efficiency [33,34]. For example, the number of fruits produced by individual plants increases fruit removal and seed dispersal by visually oriented bird frugivores [25,35,36,37,38]. At the fruit level, fruit size sets the upper limit on the size of fruits that a particular bird species can ingest [33,34,38]. Moreover, to increase the efficiency of nutrient intake, birds tend to maximize pulp ingestion by selecting bigger fruits, fruits with less or smaller seeds, or a combination of fruit and seed traits resulting in greater pulp to seed ratio [35,39,40]. However, plant traits important for the interaction with frugivores and the occurrence and relative abundance of frugivore species typically show variations among populations or between years, depending, for example, on environmental conditions and resource levels that influence plant reproductive investment and consequently the interaction between plants and their seed dispersers [25,41,42].

On the other hand, the seasonal and spatially grouped production of fruits that contain nutritious pulp and seeds also attracts antagonistic pulp eaters and seed predators during their development and ripening [10,43,44]. Species damaging fruits during the predispersal phase are mostly insects with specialized feeding habits and life cycles [43,45]. Predispersal seed predators also show preferences for some plant traits, such as the number of fruits produced and fruit characteristics related to larval survival and performance [11,44,46]. Hence, the evolution of fruit and seed characteristics favored by vertebrate dispersers would depend on the magnitude and direction of selection pressures exerted by each seed predator species on these traits [11,39,47]. Levels of fruit loss to predispersal seed predators are a direct cost to plants because they reduce the number of healthy fruits available for dispersal [10,43,44]. In addition, fruits damaged by insects favor the introduction of microbes that affect fruits and seeds [44,48], and vertebrate frugivores avoid feeding on unhealthy and damaged fruits [49,50,51]. Therefore, high rates of predispersal seed predation could negatively impact the number of ripened fruits available for dispersal by vertebrates [11]. One mechanism found in long-lived woody plants to reduce the impact of predispersal seed predators is the irregular and synchronous production of large seed crops within a population, which increases reproductive output through the satiation of predators [2,52,53], although a high annual variation in seed production might have the disadvantage of simultaneously satiating mutualistic vertebrate dispersers [9].

In this study, we examine the spatial and temporal variation in the interactions between plants, seed-dispersing birds, and three arthropod species that attack cones during the predispersal period in Spanish juniper (*Juniperus thurifera* L.). Spanish juniper produces fleshy cones (galbulae; equivalent to fleshy fruits) that are damaged by specialized arthropods which feed on cone pulp and seeds during the development and ripening period and before seeds are dispersed by vertebrate frugivores. We have previously documented population-level variations in plant traits and the incidence of antagonistic predispersal arthropods at a broad geographical scale and preferences shown by these arthropods for different plant traits during a year of high cone production at a local scale [54,55]. Here, we further explore the interaction between cone damaging arthropods and Spanish juniper and expand our previous studies estimating seed dispersal by bird frugivores, investigating the relationship between the activity of bird frugivores and plant traits and the potential negative effect of the incidence of arthropods on the amount of bird frugivory. Because seed production in the Spanish juniper exhibits large inter-annual fluctuations [55,56], we examined the interactions among juniper trees, predispersal seed predators, and seed-dispersing birds during one reproductive season in three populations located within the main distribution range of Spanish juniper in the Iberian Peninsula and during three reproductive seasons in one of these populations to assess the spatial and temporal variation in these interactions and to test whether variation in the interactions is consistent with variation in plant traits among populations and years. Specifically, we quantified the intensity of cone damage by three arthropod species, the extent of seed dispersal by birds, and characterized plant traits important for the interactions (i.e., cone crop and cone and seed traits) for each population and year. Then, we examined whether the spatial or temporal variation in individual plant traits influence the levels of predispersal seed damage by arthropods and seed dispersal by birds. We also tested for negative effects among arthropod species and whether they negatively affect the activity of bird frugivores. Finally, we evaluated whether arthropod preferences for different plant traits are similar or opposite to those preferred by dispersers, to test for potential correlated or conflicting selection pressures for those traits in some populations or years.

## 2. Materials and Methods

### 2.1. Natural History of the System

The Spanish juniper is a long-lived dioecious tree endemic to the western Mediterranean basin, with its most important populations growing under a continental Mediterranean climate in Spain and Morocco, and smaller populations in France, Italy, and Algeria. Female flowers are pollinated at the end of the winter, immature cones grow until reaching their final size by late summer, and then mature for over a year, ripening in October of the second year after pollination [57]. During the long growing and maturation period, cones are damaged by a variety of arthropods at different stages of development [55,58]. In the Iberian Peninsula, cones are mainly attacked by three arthropod groups: Mites, *Trisetacus quadrisetus* (Acarina, Phytoptidae) [57]; chalcid wasps, *Megastigmus thuriferana* (Hymenoptera, Torymidae) [59]; and two moth species, *Mesophleps oxycedrella* (Lepidoptera, Gelechiidae) and *Pammene juniperana* (Lepidoptera, Tortricidae) [57]. Mites enter the cones at the beginning of their development period and use the seeds as growth chambers. Seeds used by mites show a characteristic fluted seed wall and a deformed, elongated tip [57,58]. Chalcid wasps are seed predators that oviposit one egg inside the seeds near the end of the growing phase (mid- to late-summer), before the seed coat hardens. Larvae develop with the seeds for nearly a year, emerging as adults and leaving a characteristic circular exit hole [57,58]. Moths oviposit usually one egg on the cone’s surface during the growing and maturation period. Larvae develop feeding on the pulp, although they sometimes bore or use the seeds, leaving the cone during the ripening phase to pupate in the soil [58]. Damaged cones present galleries with fecal pellets from the larva and an exit hole on the cone surface. Ripe cones are mostly dispersed by specialized bird dispersers (thrushes, *Turdus* spp.) and generalist carnivores (red fox, *Vulpes vulpes* and stone marten, *Martes foina*) during autumn and early winter [60,61,62].

### 2.2. Study Areas

We studied three Spanish juniper populations in the central part of the Iberian Peninsula: Sigueruelo (41°10′ N, 3°39′ W, 1140 m.a.s.l., Segovia), Judes (41°7′ N, 2°11′ W, 1250 m.a.s.l., Soria), and Buenache de la Sierra (40°80′ N, 1°57′ W, 1360 m.a.s.l., Cuenca). The climate is continental Mediterranean in these regions, with mean annual temperatures of 10–11 °C and mean annual precipitation from 590 to 740 mm, with a 2-month long drought in summer. Lithology is calcareous in the three sites (see [54] for the location and further characteristics of the studied populations).

### 2.3. Variation in Plant, Cone, and Seed Traits

We estimated cone production in each of the three populations during autumn (late November) in 2007, and during two more years in Buenache (2008 and 2010). In each population, we chose 54–59 female trees at random while walking through the forest, and estimated cone production as the mean number of ripened cones counted per square meter using a 25 × 25 cm quadrat (*n* = 4–8 per plant) placed on the plant surface all around the canopy [41,63].

To characterize cone traits in each population and year, we collected ten sound cones without signs of arthropod damage to characterize cone traits. For each cone, maximum length and width were measured in the laboratory to the nearest 0.01 mm with digital calipers and averaged to calculate cone diameter. Cones were oven-dried for over 36 h at 60 °C and pulp mass and total seed mass were weighted to the nearest 0.01 mg with a digital scale. The total number of seeds from each cone was counted.

### 2.4. Variation in Cone Damage by Arthropods

To quantify cone damage by arthropods for each population and year, we collected 30 additional ripe cones from around the canopy of the same trees for which we had estimated cone production and measured cone traits. Cones were inspected and opened in the laboratory under a dissecting microscope to detect signs of damage by arthropods [55,57]. For each tree, we calculated the number of cones damaged by mites, chalcid wasps, and moths relative to the total number of fruits examined.

### 2.5. Variation in Seed Dispersal by Birds

During late November, we counted the number of thrush droppings underneath the canopy of each tree as an estimate of seed dispersal by these bird species for each plant. These droppings are easily identified due to their size and content of cone remains and seeds [41,61]. Dropping abundance under the tree canopy is considered an indirect estimate of the cumulative cone removal by thrushes, assuming that consumption is proportional to the time spend by birds on each plant [41,64]. Individual plants in populations with lower disperser activity normally had more cones remaining on the tree after the dispersal period, so this index can also be considered as a measure of tree dispersal success [41,61].

### 2.6. Numerical Analyses

#### 2.6.1. Spatial and Temporal Variation

We tested for spatial and temporal variation using the following parameters: number of cones produced, cone and seed traits, cone damage levels by each arthropod, and seed dispersal by birds (Table S1). We used general or generalized linear models including the three populations surveyed in one reproductive season as a factor predictor in the models (spatial variation). We also built similar models using the three years surveyed in the Buenache population as a predictor in the models (temporal variation). Error structures used to fit models varied depending on the distribution of the explanatory variables. Crop size and seed dispersal by birds were modeled with a negative binomial error structure. Pulp mass and number of seeds per cone were fitted using a Gaussian error structure. We did not used cone diameter and total seed mass because of the high correlations between both traits and between each variable and pulp mass (*r* > 0.52, *p* < 0.001; in all cases). Models for cone damage by arthropods used a binomial error. We checked for overdispersion in models for damage rates, and modeled overdispersion as observation-level random effects [65]. The included random effect was tree number (i.e., a discrete categorical factor to increase the spread of the distribution [65]). We estimated marginal means and made *a posteriori* pairwise comparisons (based on *Z* or *t* tests, depending on model distribution) among groups (i.e., populations or years within a population) using the emmeans package [66] in R 4.3.3 (R Development Team, Vienna, Austria). Models were fitted using base R packages and the lme4 package [67].

#### 2.6.2. Interactions among Plants, Arthropods, and Birds

We assessed the relationships between plant traits, cone damage by arthropods, and seed dispersal by birds for each year and population using piecewise structural equation models (piecewise SEMs, [68]). Piecewise structural equation modeling allows for the analyses of a set of hypothesized causal relationships, which are translated to a set of structured equations that are solved separately, and thus they can accommodate non-normal distributions, hierarchical structures, and smaller sample sizes [68,69].

We first built an *a priori* full piecewise SEM by linking four models, using previous information on this system [55,57,63]. We hypothesized that crop size and cone traits affect cone damage by arthropods, so we connected crop size, pulp mass, and number of seeds per cone to the incidence of each arthropod. Each arthropod species attacks cones at different stages of development, so the presence of one species could interfere with cone use by other species [55]. Because mites enter the cones early during cone development, we included paths from mite to chalcid wasp incidence and to moth incidence, and from chalcid wasps to moths, to test the potential interference between them. We fitted binomial generalized linear mixed models for the incidence of each arthropod species. Mixed models included an observation-level random effect to model overdispersion. Seed-dispersing birds are usually attracted to plants that produce more fruits or berry-like cones [37,41,64] and show preferences for different fruit traits, such as fruit diameter, amount of pulp, and seed number or size, depending on fruit characteristics [18,38,40,70]. Therefore, we included paths from crop size, pulp mass, and seed number to seed dispersal by birds. Birds also tend to avoid or reject unhealthy or infested fruits [44,49,50], so we connected the incidence of each arthropod to seed dispersal by birds. Models for seed dispersal by birds were fitted using generalized linear models with a negative binomial error structure. Predictor variables in all models were standardized to a zero mean and unit variance.

We linked the four generalized models to build a piecewise SEM for each population and year using the piecewiseSEM package [68]. Initial models were simplified by sequentially removing non-significant terms and using Akaike’s information criterion to obtain the most parsimonious models. We assessed the goodness-of-fit of the best piecewise SE models using the Shipley’s test of directed separation on Fisher’s C statistic that follows a ꭕ^2^ distribution. This test evaluates that there are no missing paths between unconnected variables, and a Fischer’s C statistic with *p* > 0.05 indicates and adequate model fit to the data [69].

## 3. Results

### 3.1. Crop Size and Cone Traits

**Figure 1:**
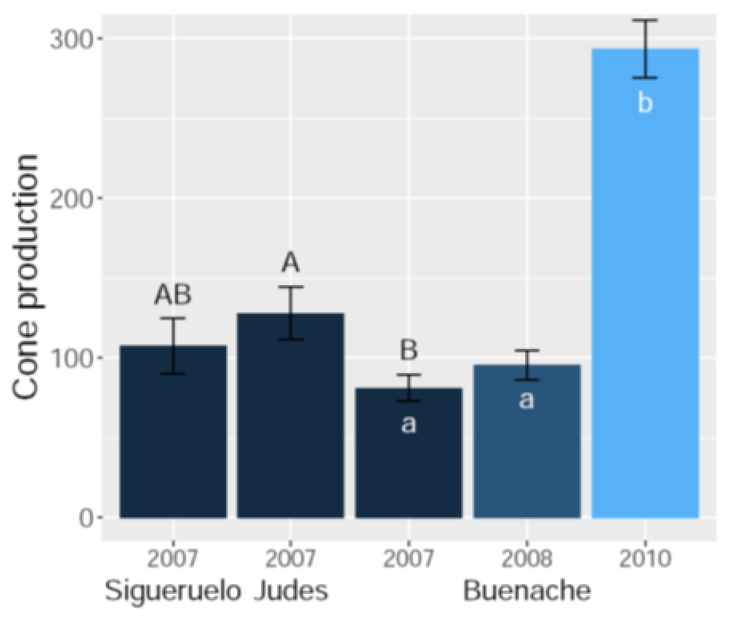
Number of cones produced (mean ± SE number of cones per m^2^) in three Spanish juniper populations in one year (2007) and during three years (2007, 2008, and 2010) in the Buenache population. Different letters indicate significant differences (uppercase letters: between-population pairwise comparisons; lowercase letters: temporal pairwise comparisons for the Buenache population).

Cone production at the population level in 2007 was lower in Buenache than in Judes (comparison of estimated marginal means: *Z* = 3.0, *p* = 0.008; Figure 1), while cone production in Sigueruelo was intermediate between the other two populations (Z < 1.8, *p* > 0.15, for both contrasts; Figure 1). On the other hand, crop size in Buenache was similar during 2007 and 2008 (Z = 1.4, *p* = 0.36; Figure 1), but substantially greater in 2010 (Z > 9.6, *p* < 0.001, for contrasts between 2007 and 2010, and between 2008 and 2010; Figure 1). Cone production in 2010 was consistent with a mast year in this juniper species with high inter-annual fluctuations in seed production [55,71].

Cone traits in 2007 showed some differences among the three populations. Trees produced cones with similar amount of pulp in Sigueruelo and Buenache (*t* = 1.5, *p* = 0.28; Table 1), but cones produced in Judes had less pulp compared to those in the other two populations (*t* > 4.2, *p* < 0.001, for both comparisons; Table 1). However, the average number of seeds per cone did not differ among the three populations (*t* < 1.7, *p* > 0.21, for the three pairwise comparisons; Table 1). Cone characteristics also differed among the three years in Buenache. Cones produced in 2007 had more pulp and less seeds than those produced in 2008 and 2010 (*t* > 2.9, *p* < 0.011, for all contrasts; Table 1). The amount of pulp was similar for the cones produced in 2008 and 2010 (*t* = 1.3, *p* = 0.40; Table 1), although cones produced in 2010 had more seeds per cone than those produced in 2008 (*t* = 2.8, *p* = 0.016; Table 1).

### 3.2. Cone Damage by Arthropods

**Figure 2:**
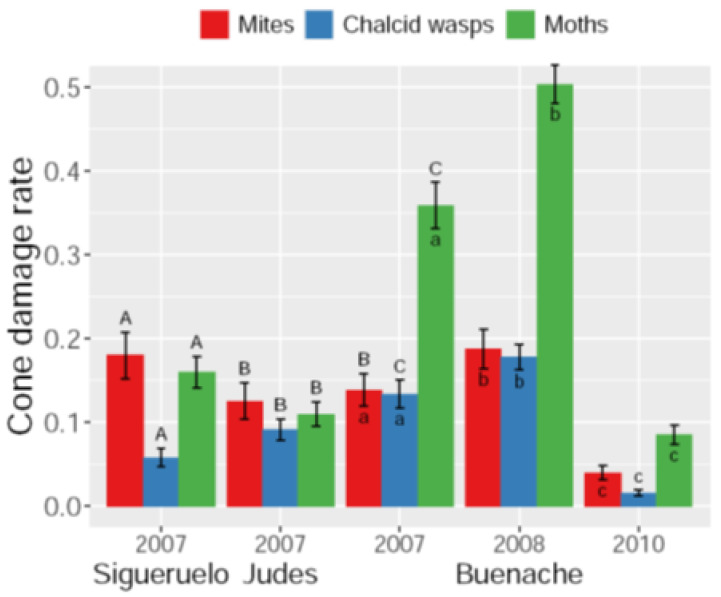
Cone damage rates (± SE) by three predispersal pulp and seed predators in three Spanish juniper populations in one year and during three different years in the Buenache population. Different letters indicate significant differences between groups within each arthropod species (uppercase letters: pairwise comparisons among the three populations; lowercase letters: temporal pairwise comparisons for the three years in the Buenache population).

Overall, cone damage by arthropods in 2007 was higher in Buenache (59.3 ± 2.8%) than in the other two populations (Sigueruelo: 38.6 ± 2.9%, Judes: 31.4 ± 2.4%). This difference was mainly due to the higher level of cone damage by moths in Buenache compared to the other two populations (Z > 12.8, *p* < 0.001, in both cases; Figure 2), and to a lesser extent, to higher seed predation by chalcid wasps (Z > 3.8, *p* < 0.001, in both cases; Figure 2). Sigueruelo showed a higher rate of cones damaged by mites compared to the other populations (Z > 2.9, *p* < 0.008, for both contrasts; Figure 2). Cone damage rates by the three arthropods showed high interannual fluctuations in Buenache (year 2007: see above; 2008: 86.9 ± 1.3%; 2010: 13.7 ± 1.3%). Damage rates by mites, chalcid wasps, and moths increased from 2007 to 2008, but were significantly reduced in the mast year of 2010 (Z > 3.2, *p* < 0.004, for all pairwise comparisons; Figure 2).

### 3.3. Seed Dispersal by Birds

**Figure 3:**
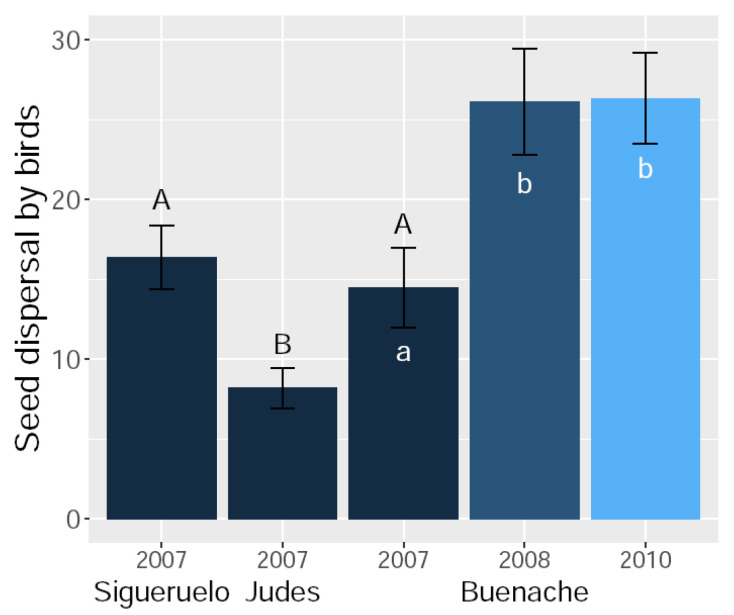
Mean number of thrush droppings (± SE) under the canopy of each tree in three Spanish juniper populations during one dispersal season and for two additional seasons in the Buenache population. Different letters indicate significant differences (uppercase letters: between-population pairwise comparisons; lowercase letters: temporal pairwise comparisons for the Buenache population).

Seed dispersal by thrushes differed among populations, and among years within the same population (Figure 3). Seed dispersal in 2007 was lower in Judes than those in Sigueruelo and Buenache (Z > 2.9, *p* < 0.009, in both cases; Figure 3), which showed similar seed dispersal rates (Z = 0.6, *p* = 0.80; Figure 3). Seed dispersal rates in Buenache were higher in 2008 and 2010 compared to that in 2007 (Z > 3.1, *p* < 0.005, in both cases; Figure 3), although dispersal rates did not differ between 2008 and 2010 (Z = 0.1, *p* = 0.99; Figure 3).

### 3.4. Variation in the Interactions among Plants, Arthropods and Birds

The most parsimonious SE models showed a good fit to the data in Sigueruelo in 2007 (Fisher’s C = 7.7, *p* = 0.65) and in Buenache during the three years (2007: Fisher’s C = 20.9, *p* = 0.40; 2008: Fisher’s C = 4.5, *p* = 0.61; 2010: Fisher’s C = 22.9, *p* = 0.19), whereas the fit was lower for Judes in 2007 (Fisher’s C = 14.1, *p* = 0.08).

**Figure 4:**
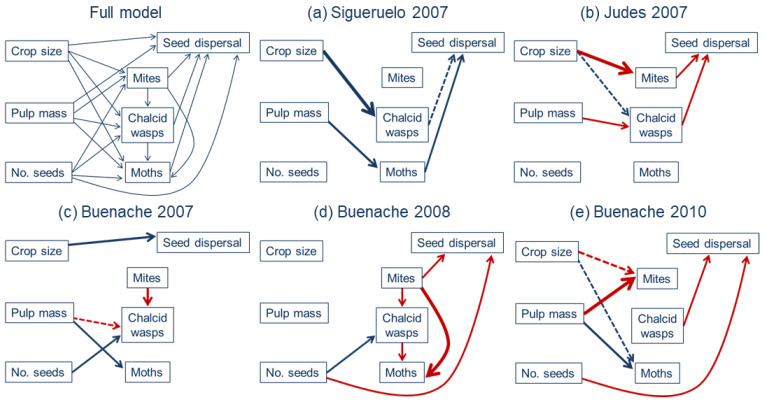
Full and best piecewise structural equation models for the relationships among plant traits (crop size, pulp mass, and number of seeds per cone), cone damage rates by three arthropods, and seed dispersal by thrushes in three Spanish juniper populations (Sigueruelo, Judes, and Buenache) during 2007 and in two additional years (2008 and 2010) in the Buenache population. Positive effects are indicated with solid blue (*p* < 0.05) or dashed blue (*p* < 0.1) arrows and negative effects with solid red (*p* < 0.05) or dashed red (*p* < 0.1) arrows. The width of the arrows is proportional to the path coefficients values (Table S2).

In Sigueruelo 2007, chalcid wasps preferred trees producing more fruits, whereas moths preferred those producing cones with more pulp (Figure 4, Table S2a). Crop size and pulp mass did not have a direct effect on seed dispersal by birds, but birds dispersed more seeds from trees having more cones damaged by chalcid wasps and moths (Figure 4). In contrast, in Judes 2007, seed dispersal was negatively influenced by the incidence of mites and chalcid wasps (Figure 4). The incidence of mites was negatively influenced by tree crop size, and seed predation by chalcid wasps was positively correlated with crop size and negatively with pulp mass (Figure 4, Table S2b). In Buenache, models showed interannual differences. In 2007, seed dispersal by birds increased in trees that produced more cones and was not affected by the incidence of arthropods (Figure 4). Seed predation by chalcid wasps was negatively affected by the incidence of mites at the same tree, and predation rates increased in trees producing cones with less amount of pulp and more seeds. Moths showed a preference for trees producing cones with more pulp (Figure 4, Table S2c). Models for Buenache during the other two years (2008 and 2010) were not similar, although birds avoided trees producing cones with more seeds in both years and seed dispersal was negatively influenced by the incidence of one arthropod species (mites in 2008 and chalcid wasps in 2010; Figure 4). In 2008, chalcid wasps preferred trees with more seeds per cone and avoided trees with higher levels of cones damaged by mites. In addition, cone damage by moths was negatively affected by the incidence of both mites and chalcid wasps (Figure 4, Table S2d). In 2010, when tree crop size was higher, the incidence of mites was negatively related to tree crop size and correlated positively with pulp mass. On the other hand, cone damage by moths increased in trees producing more cones with more pulp (Figure 4, Table S2e).

## 4. Discussion

Spanish juniper populations showed important spatiotemporal variations in plant traits as well as in the incidence and activity of specific seed predator arthropods and bird frugivores. The interactions between this juniper species and their cone damaging arthropods and seed dispersing birds were dynamic in space and time, being largely determined by temporal fluctuations in cone abundance.

### 4.1. Variation in Plant Traits and Interacting Animal Species

Cone crop size showed great temporal fluctuations. Spanish juniper, as other juniper species, shows high inter-annual variation in seed production [9,41,62,72,73], producing significant seed crops once or twice every 10 years [55,56]. Spatial and temporal variability also affected cone traits at the population and individual levels. Differences in reproductive traits can be partly due to variation in resource levels and plant reproductive investment [18,35,74]. For example, larger crops during years of favorable environmental conditions in Spanish juniper are associated with a higher number of seeds being set, presumably due to efficient pollination and maternal effects [54]. Nevertheless, some cone and fruit traits can have a relatively high heritability, suggesting a potential response to selection [38,75].

Differences in rates of cone damage by each arthropod species were not so pronounced among the three populations. The main difference was the higher impact of moths in the Buenache population. Spatial variation in these antagonistic interactions might be relatively consistent during years of medium to low population-level crop sizes. For example, mites tend to be less common in juniper woodlands under more arid conditions [54,76,77], and the incidence of chalcid wasps decreases in populations with higher annual precipitation [54,78]. However, the most notable variation in levels of cone damage by the three arthropod species was between years within the same population. This is consistent with the negative effect of crop size on seed predation rates through a satiation effect of predators in mast seeding plants [9,52,55,72].

The activity of frugivorous thrushes varied among the three populations and between years. Crop size is usually an important determinant of fruit removal and seed dispersal by birds [37,38]. However, several extrinsic factors, such as habitat and landscape structure, availability of perching sites, and the abundance of other fruiting plant species, can also shape bird-mediated seed dispersal [38,41,63]. We observed temporal variation in seed dispersal within the same population, increasing during a year of large seed production, although the amount of frugivory did not differ from the other year with lower cone production. This suggests a potential satiation effect on local avian dispersers [9,15,79]. However, we quantified seed dispersal by birds during late autumn or early winter, which could have somewhat underestimated the cumulative cone removal by migratory thrushes. Migratory thrushes show greater ability in tracking fruits at different scales than sedentary thrushes, and their abundance tends to increase from autumn to winter during large crops [79]. Therefore, despite the potential satiation of thrushes during the mast-seeding year, the amount of frugivory should probably be higher than our estimation [73].

### 4.2. Spatiotemporal Variation in Plant–Animal Interactions

We found relatively heterogeneous responses of predispersal seed predator arthropods and seed-dispersing birds to plant traits in different populations and years within a population. However, our results show patterns consistent with previous findings in this system and similar systems. The three arthropod species were influenced by interindividual variation in plant traits. Mites, the less mobile species, were negatively affected by larger crop sizes through a satiation effect at the scale of individual trees; a response observed in specialized seed predators with low dispersal capacity [53,55,80]. The incidence of mites was also lower in trees with a greater amount of pulp in cones during the masting year. Because mites enter the cones and seeds early during the development period, the negative relationship between this cone trait and the incidence of mites (a connection between variables strongly suggested by the Shipley’s test to increase model fit) could be an indirect indicator of early cone characteristics (e.g., size of first-year unripe cones and fully ripened cones is positively correlated at individual trees; E. Rodríguez personal communication), or other plant traits such as plant reproductive investment. Chalcid wasps responded to tree-level crop size and cone traits in different populations and years, except during the masting year when the incidence of this wasp species was low (see also [55]). Chalcid wasps preferred trees that produced more cones and those having cones with more seeds or less pulp. Plants producing more fruits usually attract mobile insect frugivores through visual and olfactory cues, thus crop size tends to correlate positively with the intensity of predispersal seed predation [44,45,54]. Moreover, female chalcid wasps insert the ovipositor into the cone to reach the seeds and lay eggs, so cones with more seeds and less pulp could optimize oviposition and larval performance [54,81]. On the other hand, moths oviposit on cones and larvae develop by feeding mostly on cone pulp [57]. Our results for different populations and years indicate that moths consistently preferred trees producing cones with higher amount of pulp, which would provide more resources for their larvae, likely improving their survival and performance [44,55].

Crop size and fruit characteristics are the main plant-level traits explaining interindividual variation in the number of seeds dispersed by vertebrate frugivores [33,37,38]. We detected direct preferences for these plant traits by seed-dispersing thrushes in the Buenache population during the three study years. Thrushes preferred trees with more cones the year that the cones produced had more pulp and less seeds, whereas they favored trees producing cones with less seeds the years that cones had less pulp and contained more seeds. This is consistent with increased foraging efficiency by thrushes, so that they maximize the ingestion of nutritious pulp relative to the intake of seeds [40,44,82]. We did not detect direct relationships between plant traits and seed dispersal by thrushes in the other two populations. Bird frugivory in Sigueruelo was positively associated with the incidence of moths and chalcid wasps, although the incidence of both insects was relatively low. Cone damage by moths did not affect seed dispersal by thrushes in other populations and years (see below), and thrushes do not seem to actively feed on cones infested by moths because larvae leave ripened cones during early autumn to pupate in the soil [57,58]. In addition, thrushes avoid feeding on juniper cones damaged by chalcid wasps [49]. Therefore, the positive correlations between frugivory by thrushes and cone damage by both insects suggest positive indirect relationships between the amount of frugivory and the plant traits preferred by each insect species (i.e., trees with larger crops and cones with a higher amount of pulp).

### 4.3. Interactions among Antagonistic and Mutualistic Animals

We detected interference or competition between arthropod species in only one population (Buenache) during the two years of low cone production. During these two reproductive seasons, cone damage levels by the three arthropods were the highest compared to other populations and years. The incidence of chalcid wasps at individual trees was negatively affected by the incidence of mites, presumably because mites reduce the number of cones available for ovipositing female chalcid wasps [54,55]. Furthermore, we have previously found an increase in the incidence of chalcid wasps after experimentally reducing the incidence of mites in this juniper species [57]. The next year, when cone damage levels increased for the three arthropod species, there were negative interactions between the three arthropods according to their life cycle. The incidence of mites had a negative effect on the incidence of chalcid wasps and moths, and the incidence of chalcid wasps was negatively associated with the incidence of moths. Therefore, competition among seed predators increased during years of low cone production and high rates of cone damage by each arthropod species, when the proportion of cones attacked by more than one arthropod is higher [45,55,83].

Our results suggest that predispersal seed predators can sometimes negatively affect the activity of bird frugivores [49,50,51]. Mites colonize cones at an early stage of development, deforming the seeds, and grown cones are usually smaller in size or have a smaller amount of pulp. Moreover, chalcid wasps emerge as adults leaving the cone by an exit hole before thrushes start to feed on ripe cones. Thus, cones damaged by both arthropods have lower nutritional value and are likely to have a worse health status. We found that thrushes seem to avoid feeding on trees highly attacked by mites or chalcid wasps in two study populations during different years. However, despite the fact that the incidence of moths was particularly high in the Buenache population, we did not detect an effect of moth infestation on frugivory by thrushes. This is unexpected because the nutritional value of cones used by moth larvae should be lower and contain fecal pellets from the larva, although that could depend on the amount of pulp consumed by the larva before leaving the cone. Nonetheless, it is unclear whether thrushes consume cones damaged by moths.

### 4.4. Outcomes of Antagonistic and Mutualistic Interactions on Spanish Juniper

The assemblage of antagonistic predispersal seed predators and mutualistic avian dispersers exerted selective pressures on Spanish juniper traits. For example, we found that thrushes preferentially feed on cones having less seeds and female chalcid wasps prefer to oviposit in cones with more seeds. This suggests correlational selection between seed predators and dispersers, so female juniper trees should produce cones with less seeds to improve fitness at the stage of seed dispersal. However, selective pressures exerted by chalcid wasps and thrushes were not consistent among populations and years. Moreover, seed number in Spanish juniper cones seem to respond plastically to the available resources and maternal effects [54,57], although this plastic investment could be adaptive if the production of more viable seeds increases fitness at later stages [74,84]. At a larger geographical scale, Spanish juniper populations in North Africa produce cones with mostly a single large seed, which seem to be a response to the lower intensity of predispersal seed predation by arthropods (and perhaps to differences in the assemblage of seed dispersers) compared to the Iberian populations [77]. Iberian Spanish juniper populations interact with similar predispersal seed predators and dispersers, and there were not consistent differences in the direction and intensity of selection pressures they exerted, so populations do not seem to be geographically structured into selection mosaics [5,30,85].

The most consistent pattern in the interaction among these species was the response of seed predators and dispersers to large temporal fluctuations in cone production by the juniper trees [55,79]. As in other woody perennials, junipers produce irregular and synchronous cone crops as an evolutionary mechanism to reduce seed predation by specialized predispersal seed predators through a satiation effect during large crops [9,55]. However, high supra-annual variations in cone production by junipers also leads to the satiation of local avian dispersers [9,15,73,79]. Despite the disadvantage of satiating bird frugivores, juniper species tend to favor the avoidance of specific seed predators [9,52]. Therefore, large crops reduce seed loss by predispersal seed predators and increase the number of viable seeds dispersed, resulting in higher plant reproductive success [9,15,52]. Nevertheless, some trees produce cones between large seed production events, and despite fewer viable seeds being set [54,55] and a lower abundance and activity of avian frugivores, some seeds might survive and successfully recruit. Since satiation of specialized seed predators occurs at local scales, juniper trees reproducing synchronously at scales according to the mobility of seed predators during low population-level seed crops would be favored to improve reproductive success in years between large crop events [52,55].

## Figures and Tables

**Table 1:** Pulp mass and number of seeds per cone (mean ± SE) for cones produced by Spanish juniper trees in three populations during 2007, and during three years in the Buenache population.

Population	Year	Pulp Mass (g)	No. Seeds per Cone
Sigueruelo	2007	0.29 ± 0.01	3.24 ± 0.08
Judes	2007	0.24 ± 0.01	3.08 ± 0.09
Buenache	2007	0.31 ± 0.01	3.02 ± 0.10
	2008	0.28 ± 0.01	3.44 ± 0.10
	2010	0.26 ± 0.01	3.82 ± 0.09

## Data Availability

The data presented in this study are available in the Supplementary Materials.

## Supplementary Materials

The following supporting information can be downloaded at: https://www.mdpi.com/article/10.3390/insects15080620/s1, Table S1: Excel file with the data presented in this study, Table S2: Summary of the best piecewise structural equation model coefficients for several Spanish juniper populations and years.
