# Supplementary material for: Spatial and Temporal Variation in the Antagonistic and Mutualistic Interactions among Seed Predator Arthropods, Seed-Dispersing Birds, and the Spanish Juniper
Source: Insects. 2024 Aug 18;15(8):620. doi: 10.3390/insects15080620 (PMC11354789; doi:10.3390/insects15080620)
Supplement: Supplementary file 1 [file insects-15-00620-s001.zip › Table S2.pdf]

Table S2. Summary of the best piecewise structural equation model coefficients for several Spanish juniper populations and years.

Population	Response	Predictor	Estimate	SE	*p*
(a) Sigueruelo 2007					
	Seed dispersal	Chalcid wasps	0.199	0.113	0.079
	Seed dispersal	Moths	0.243	0.114	0.033
	Chalcid wasps	Crop size	0.553	0.202	0.006
	Moths	Pulp mass	0.373	0.149	0.012
(b) Judes 2007					
	Seed dispersal	Mites	-0.359	0.140	0.010
	Seed dispersal	Chalcid wasps	-0.268	0.128	0.036
	Mites	Crop size	-0.675	0.291	0.020
	Chalcid wasps	Crop size	0.320	0.137	0.020
	Chalcid wasps	Pulp mass	-0.261	0.159	0.100
(c) Buenache 2007					
	Seed dispersal	Crop size	0.565	0.132	<0.001
	Chalcid wasps	Mites	-0.460	0.166	0.005
	Chalcid wasps	Pulp mass	-0.273	0.151	0.071
	Chalcid wasps	No. Seeds	0.299	0.146	0.041
	Moths	Pulp mass	0.315	0.135	0.020
(d) Buenache 2008					
	Seed dispersal	Mites	-0.356	0.114	0.002
	Seed dispersal	No. Seeds	-0.246	0.108	0.023
	Chalcid wasps	Mites	-0.224	0.080	0.005
	Chalcid wasps	No. Seeds	0.252	0.066	<0.001
	Moths	Mites	-0.708	0.067	<0.001
	Moths	Chalcid wasps	-0.368	0.053	<0.001
(e) Buenache 2010					
	Seed dispersal	Chalcid wasps	-0.271	0.117	0.021
	Seed dispersal	No. Seeds	-0.327	0.113	0.004
	Mites	Crop size	-0.433	0.232	0.063
	Mites	Pulp mass	-0.608	0.218	0.005
	Moths	Crop size	0.282	0.144	0.051
	Moths	Pulp mass	0.413	0.144	0.004
